# Prolonged Ketosis in a Patient With Euglycemic Diabetic Ketoacidosis Secondary to Dapagliflozin

**DOI:** 10.1177/2324709617710040

**Published:** 2017-05-24

**Authors:** Shreya Pujara, Adriana Ioachimescu

**Affiliations:** 1 Emory University, Atlanta, GA, USA

**Keywords:** euglycemic diabetic ketoacidosis, sodium-glucose cotransporter 2 inhibitors, persistent, prevention

## Abstract

Since the approval of sodium-glucose cotransporter 2 (SGLT2) inhibitors by the US Food and Drug Administration for type 2 diabetes, there have been several reports of euglycemic diabetic ketoacidosis in patients using this class of medication. We present a case of euglycemic diabetic ketoacidosis where ketonemia and glucosuria persisted well beyond the expected effect of dapagliflozin. Our patient is a 50-year-old woman with type 2 diabetes since age 35 who was taking metformin and dapagliflozin. She presented with fatigue, constipation, and 3 days of reduced oral intake. Laboratory data indicated anion gap acidosis, ketonemia, severe hypokalemia, and minimally elevated blood glucose. She was treated with sliding scale short-acting insulin and electrolyte replacement until hospital day 6, when endocrinology was consulted. An insulin drip was initiated due to persistent ketonemia and reopening of the anion gap, despite improved oral intake and normoglycemia. On stopping the insulin drip on day 9, the β-hydroxybutyrate increased again. It finally stabilized within normal range with the initiation of basal subcutaneous insulin. This case indicates that clinical effects of dapagliflozin persist much longer than the reported half-life of 12.9 hours would predict. To prevent this potentially dangerous complication, patients taking SGLT2 inhibitors who become ill should discontinue the medication, undergo ketone evaluation, and start basal insulin, if ketones are positive. In addition, patients should be educated to stop their SGLT2 inhibitor at least 1 week prior to elective procedures.

## Introduction

Sodium-glucose cotransporter 2 inhibitors (SGLT2) are the newest class of oral agents to receive US Food and Drug Administration (FDA) approval for the treatment of type 2 diabetes (T2DM). SGLT2 inhibitors currently approved by the FDA include canagliflozin, dapagliflozin, and empagliflozin as well as various combination drugs (Table 1). The enthusiasm this class of drugs has been greeted with stems from the benefits associated with SGLT2 inhibitors. They include decrease in A1c by 0.5% to 1%, reduction in insulin doses, modest weight loss, and improved systolic and diastolic blood pressure.^1^ In addition, the EMPA-REG OUTCOME trial showed a reduction in all-cause and cardiovascular mortality with empagliflozin.^2^ Also, a post hoc analysis of a study on dapagliflozin in type 2 diabetics with moderate renal impairment showed improved albuminuria and delayed progression to severe renal failure.^3^

**Table 1.** SGLT2 Inhibitors Approved in the United States.

Generic Name	Brand Name	Company Producing	Date of FDA Approval
Canagliflozin	Invokana	Janssen	March 29, 2013
Dapagliflozin	Farxiga	Astrazeneca	January 8, 2014
Empagliflozin	Jardiance	Boehringer Ingelheim	August 1, 2014
Canagliflozin + metformin	Invokamet	Janssen	August 8, 2014
Dapagliflozin + metformin	Xigduo	Astrazeneca	October 29, 2014
Empagliflozin + linagliptin	Glyxambi	Boehringer Ingelheim	January 30, 2015
Empagliflozin + metformin	Synjardy	Boehringer Ingelheim	August 29, 2015
Canagliflozin + metformin	Invokamet XR	Janssen	September 20, 2016

Abbreviations: SGLT2, sodium-glucose cotransporter 2; FDA, US Food and Drug Administration.

The popularity of SGLT2 inhibitors is understandable considering the paucity of oral diabetic drugs that promote both weight loss and reduction of insulin needs. Endocrinologists and internists alike have increasingly prescribed this class of drugs as to avoid initiation of insulin or escalation of insulin doses. With more patients using SGLT2 inhibitors, reports of euglycemic diabetic ketoacidosis (euDKA) have emerged. While DKA can be expected with off-label use of SGLT2 inhibitors in patients with T1DM, it has also occurred in T2DM patients. Thus, the FDA posted a drug safety communication on DKA in 2015.^4^ Greater understanding of how to safely use this newest tool in our arsenal against diabetes is essential.

## Case Description

A 50-year-old African American female with T2DM since the age of 35 presented with 10 days of constipation and fatigue, as well as reduced oral intake for 3 days prior to admission. She reported discontinuing antihyperglycemic medications 2 days prior to admission. Her regimen consisted of metformin 500 mg twice daily and dapagliflozin 10 mg daily, which she was taking for a year. Her hemoglobin A1c (HA1c) at admission was 8.2%, higher compared to the previous year at which time she was taking metformin 500 mg BID and glimepiride 4 mg daily. The reason for adjustment of the regimen was weight gain. She had no history of microvascular or macrovascular complications. Family history was significant only for hypertension. Social history was negative for alcohol or tobacco use.

On initial assessment in the emergency room she was noted to be acidotic (pH on ABG of 7.34) with an elevated anion gap and β-hydroxybutyrate (bHB), severe hypokalemia, hypophosphatemia, and acute kidney injury (Table 2). Physical examination on presentation was significant for sinus tachycardia. Her temperature was 36.8°C, heart rate 104 beats per minute, respiratory rate of 18, blood pressure 163/80, oxygen saturation 100% on room air, weight 93 kg, and body mass index 26 kg/m^2^. There was no evidence of infection and viral testing, and cultures were all negative. She was briefly started on an insulin drip but this was discontinued within 1 to 2 hours due to the severity of her hypokalemia. Sliding scale Humalog for blood sugar greater than 180 was started of which she received only one dose. The highest blood glucose level was 202, on the day of admission. She required aggressive replacement of both phosphate and potassium orally and parenterally. Serum creatinine and estimated glomerular filtration rate normalized after 2 days, while appetite and food intake improved throughout the hospital stay. Although the anion gap and bHB initially improved, on day 6 of admission Endocrinology was consulted due to abnormal anion gap and bHB. At that time, the patient had been off dapagliflozin for 8 days, blood glucose levels were in the 100 to 180 mg/dL range, and urine glucose level >1000. Insulin drip was started along with dextrose infusion (D5 ½ NS + 40 mEq KCl, at 70 mL/h). The initial rate of the insulin drip was set at 0.8 U/h and titrated up to 1 U/h on day 8. The anion gap and bHB gradually improved and the insulin drip was discontinued on the morning of hospital day 9. After a few hours, the bHB rose again, which prompted initiation of insulin glargine 10 units daily. On follow-up 8 weeks later, she was transitioned back to oral agents with metformin 1000 mg BID and glimepiride 8 mg daily. Testing at that time showed negative GAD-65 and islet cell ab levels as well as a C-peptide of 1.8 ng/mL with a concomitant blood glucose of 204 mg/dL.

**Table 2.** Laboratory Data During Hospital Course.

	Na (136-144 mmol/L)	K (3.6-5.1 mmol/L)	Cl (101-111 mmol/L)	AG (2-11)	bHB (<0.3 mmol/L)	CO_2_ (22-32 mmol/L)	Phos (2.4-4.7 mmol/L)	Serum Glucose (mg/dL)	Urine Glucose (mg/dL)	Creatinine (0.4-1.0 mg/dL)	eGFR (>60 mL/min/m^2^)
Day 1	136	2.2	98	25	>8.0	13		202	>1000	1.41	50
Day 2	136	2.2	104	19		13	1.2	186^a^		1.29	56
Day 3	128	2.2	104	8		16	1.4	117^a^		0.94	>70
Day 4	136	2.9	105	10	4.0	21	2.2	164		0.77	>70
Day 5	140	3.1	105	14		21	2.4	162^a^		0.90	>70
Day 6—insulin drip started	135	3.2	100	15	4.8	20	3.0	152^a^	>1000	0.80	>70
Day 7	142	4.0	106	9	1.7	27	3.2	164^a^		0.59	>70
Day 8	142	4.1	106	7	0.5	29		233^a^	300	0.60	>70
Day 9 am—insulin drip stopped					0.1						>70
Day 9 pm	138	4.0	98	10	0.5	30		192	100	0.59	>70
Day 10	141	3.7	102	9	0.3	30		167^a^		0.52	>70

Abbreviations: bHB, β-hydroxybutyrate; eGFR, estimated glomerular filtration rate.

a Fasting glucose.

## Discussion

Our case of a patient with T2DM with euDKA persistent 8 days after discontinuation of dapagliflozin supports longer lasting effects than what was expected based on its half-life. While our patient had slight decrease of her kidney function on admission, this resolved quickly with fluid replacement. An insulin drip was necessary to correct the metabolic imbalance. Another important feature of our case was the lack of precipitating factors except for poor oral intake, which actually may have been caused by the ketonemia.

DKA is defined by the triad of hyperglycemia (>250 mg/dL), anion-gap acidosis, and increased plasma ketones. Euglycemic DKA is defined as DKA without hyperglycemia. This can be caused by SGLT2 inhibitors, with 20 reported cases between March 2013 and June 6, 2014.^4^ A controlled study on the issue of euDKA has not been done. Most reported patients had T2DM, and potential triggers were infection, trauma, reduced caloric intake, alcohol use, and reduced insulin doses. Several additional cases were subsequently reported, which are represented in Table 3. Two reports of euDKA in the United Kingdom pertained to patients with pancreatic insufficiency taking dapagliflozin.^5^ Although initially presumed to have T2DM, these patients had secondary diabetes in the context of chronic pancreatitis and distal pancreatectomy, respectively, with low levels of insulin and C-peptide and negative autoantibodies. In 2016, a case of euDKA was reported in a patient with advanced insulin-dependent T2DM who discontinued insulin therapy completely after starting empagliflozin.^6^ A case of euDKA reported in Japan was precipitated by a low carbohydrate diet in a patient whose diabetes regimen had recently been changed from glimepiride, metformin, and linagliptin to ipraglaflozin alone.^7^ A case of recurrent euDKA along with acute elevation of creatinine (from 0.6 to 1.19) was reported in a patient with a solitary kidney who underwent elective abdominoplasty.^8^ Although it initially resolved with intravenous insulin and dextrose infusion, it recurred on transition to basal plus insulin therapy, 5 days after the last dose of canagliflozin (creatinine 0.7 mg/dL). Peters et al reviewed 9 cases of euglycemic DKA, 7 in T1DM and 2 in T2DM patients.^9^ In the T1DM group, 4 had recurrences of euDKA upon rechallenge with the SGLT2 inhibitor and one had persistent ketonuria 48 hours after discontinuation of canagliflozin. Some patients were treated for their recurrent euDKA on an outpatient basis, with discontinuation of their SGLT2 inhibitor, increased insulin doses, and increased carbohydrate intake. The 2 T2DM patients reported by Peters et al were taking canagliflozin; both developed euDKA after elective surgical procedures, which resolved after intravenous administration of insulin and fluids. In one of these patients, complete metabolic recovery did not occur until 6 days after the last dose of canagliflozin.

**Table 3.** Cases of Euglycemic DKA.

Author (Year)	Number of Cases	SGLT2 Inhibitor	Diabetes Type	Precipitating Factors
Hine et al (2015)^5^	1 Male, 1 female	Dapagliflozin	Secondary diabetes	Pancreatic insufficiency
Roach and Skierczynski (2016)^6^	1 Female	Empagliflizon		
Hayami et al (2015)^7^	1 Female	Ipragliflozin	T2DM	Low carbohydrate diet
Maraka et al (2016)^8^	1 Female	Canagliflozin	T2DM	Surgery
Peters et al (2015)^9^	7 Female	Canagliflozin	T1DM	Infection, alcohol
	1 Male, 1 female	Canagliflozin	T2DM	Surgery

Abbreviations: DKA, diabetic ketoacidosis; SGLT2, sodium-glucose cotransporter 2; T2DM, type 2 diabetes mellitus.

The etiology of euDKA with use of SGLT2 inhibitor is multifactorial.^10^ SGLT2 inhibitors act on transporters in the proximal tubule and reduce reabsorption of glucose, thereby increasing urinary glucose excretion. This can promote a starvation state and ketogenesis in patients on a carbohydrate-restricted diet or in those with reduced food intake in the context of illness or perioperative state. In addition, due to improved glycemia, patients often decrease their insulin doses, which augments the risk for ketogenesis in patients with advanced β-cell dysfunction. On the other hand, SGLT2 is expressed on pancreatic α-cells, so their inhibition increases glucagon release.^11^ Finally, the renal clearance of ketone bodies may be reduced, as indicated by studies with nonselective SGLT2 inhibitor phlorizin.^12^ In summary, reduced serum glucose, reduced insulin doses, increased glucagon release, and reduced clearance of ketone bodies are contributors to euDKA in patients taking SGLT2 inhibitors.^11^

In our case of an African American patient with a diagnosis of T2DM presenting with ketoacidosis, differential diagnosis included ketosis-prone diabetes mellitus. However, this was not supported by glucose levels, which are usually significantly elevated in this entity. We attribute the euDKA diagnosis to dapagliflozin treatment and point out that glucosuria and ketonemia persisted 8 days after stopping dapagliflozin. Euglycemic DKA seems to have been triggered by reduced oral intake. The lack of continuous insulin administration early during hospitalization contributed to the persistence of DKA; however, persistent glucosuria despite minimal serum glucose elevation suggests an ongoing effect of dapagliflozin. Considering the estimated half-life of 12.9 hours for dapagliflozin, it would be expected that glucosuria would resolve 2 to 3 days after discontinuation of the drug. The glucosuria with blood glucose values largely below the normal renal threshold seen 8 days after last use of dapagliflozin in our patient suggests the effect of this medication may be longer than expected. A better understanding of the pharmacodynamics of SGLT2 inhibitors is needed.

Our case along with other reports of euDKA in T2DM raise several management issues in patients treated with SGLT2 inhibitor. First, it is likely this entity is underrecognized in the absence of significant hyperglycemia. A careful evaluation of risk factors for DKA is necessary prior to starting SGLT2 inhibitor in patients with advanced β-cell dysfunction, if acutely ill or eating poorly, and 1 week prior to surgical procedures. Initiation of basal insulin perioperatively may be necessary, especially in those with advanced β-cell dysfunction. For patients diagnosed with euDKA while taking SGLT-2 inhibitors, the following steps are essential: stopping the SGLT2 inhibitor, starting long-acting insulin (or increasing insulin doses for those already taking it), increasing carbohydrate intake (if possible), and serial monitoring of electrolytes and ketones. Importantly, short-acting sliding scale insulin may not be sufficient to correct the metabolic acidosis. While mild cases of euDKA can be treated in the ambulatory setting, patients should be admitted in case of vomiting, infection, systemic illness, inability to eat, dyselectrolytemia, or acute changes in the kidney function. When insulin drip is required, the algorithm should be individualized depending on glucose levels and a concomitant dextrose drip should be administered to maintain euglycemia. Further studies are needed to understand which patients are at risk for this complication and its therapeutic implications.
